# German *Francisella tularensis* isolates from European brown hares (*Lepus europaeus)* reveal genetic and phenotypic diversity

**DOI:** 10.1186/1471-2180-13-61

**Published:** 2013-03-21

**Authors:** Wolfgang Müller, Helmut Hotzel, Peter Otto, Axel Karger, Barbara Bettin, Herbert Bocklisch, Silke Braune, Ulrich Eskens, Stefan Hörmansdorfer, Regina Konrad, Anne Nesseler, Martin Peters, Martin Runge, Gernot Schmoock, Bernd-Andreas Schwarz, Reinhard Sting, Kerstin Myrtennäs, Edvin Karlsson, Mats Forsman, Herbert Tomaso

**Affiliations:** 1 Institute of Bacterial Infections and Zoonoses, Friedrich-Loeffler-Institut (Federal Research Institute for Animal Health), Naumburger Str. 96A, Jena D-07743, Germany; 2 Institute of Molecular Biology, Friedrich-Loeffler-Institut (Federal Research Institute for Animal Health), Südufer 10, Greifswald-Insel Riems D-17493, Germany; 3 Thüringer Landesamt für Lebensmittelsicherheit und Verbraucherschutz, Tennstedter Str. 9, Bad Langensalza D-99947, Germany; 4 Lower Saxony State Office for Consumer Protection and Food Safety, Eintrachtweg 17, Hannover D-30173, Germany; 5 Landesbetrieb Hessisches Landeslabor, Schubertstr. 60, Gießen D-35393, Germany; 6 Bayerisches Landesamt für Gesundheit und Lebensmittelsicherheit, Veterinärstr. 2, Oberschleißheim D-85764, Germany; 7 Staatliches Veterinäruntersuchungsamt Arnsberg, Zur Taubeneiche 10-12, Arnsberg D-59821, Germany; 8 Official Laboratory for Public and Veterinary Health Saxony, Leipzig, Bahnhofstr. 58-60, Leipzig D-04158, Germany; 9 Chemisches und Veterinäruntersuchungsamt Stuttgart, Schaflandstr. 3/3, Fellbach D-70763, Germany; 10 CBRN Defence and Security, Swedish Defence Research Agency (FOI), Umeå SE-90182, Sweden

## Abstract

**Background:**

Tularemia is a zoonotic disease caused by *Francisella tularensis* that has been found in many different vertebrates. In Germany most human infections are caused by contact with infected European brown hares (*Lepus europaeus*). The aim of this study was to elucidate the epidemiology of tularemia in hares using phenotypic and genotypic characteristics of *F. tularensis*.

**Results:**

Cultivation of *F. tularensis* subsp. *holarctica* bacteria from organ material was successful in 31 of 52 hares that had a positive PCR result targeting the Ft-M19 locus. 17 isolates were sensitive to erythromycin and 14 were resistant. Analysis of VNTR loci (Ft-M3, Ft-M6 and Ft-M24), INDELs (Ftind33, Ftind38, Ftind49, RD23) and SNPs (B.17, B.18, B.19, and B.20) was shown to be useful to investigate the genetic relatedness of *Francisella* strains in this set of strains. The 14 erythromycin resistant isolates were assigned to clade B.I, and 16 erythromycin sensitive isolates to clade B.IV and one isolate was found to belong to clade B.II. MALDI-TOF mass spectrometry (MS) was useful to discriminate strains to the subspecies level.

**Conclusions:**

*F. tularensis* seems to be a re-emerging pathogen in Germany. The pathogen can easily be identified using PCR assays. Isolates can also be identified within one hour using MALDI-TOF MS in laboratories where specific PCR assays are not established. Further analysis of strains requires genotyping tools. The results from this study indicate a geographical segregation of the phylogenetic clade B.I and B.IV, where B.I strains localize primarily within eastern Germany and B.IV strains within western Germany. This phylogeographical pattern coincides with the distribution of biovar I (erythromycin sensitive) and biovar II (erythromycin resistance) strains. When time and costs are limiting parameters small numbers of isolates can be analysed using PCR assays combined with DNA sequencing with a focus on genetic loci that are most likely discriminatory among strains found in a specific area. In perspective, whole genome data will have to be investigated especially when terrorist attack strains need to be tracked to their genetic and geographical sources.

## Background

Tularemia is a rare zoonotic disease caused by *Francisella tularensis*, a Gram negative, facultative intracellular, fastidious bacterium [1]. Most infections in animals and humans are caused by two *F. tularensis* subspecies, *F. tularensis* subsp. *tularensis* (Jellison type A) and *F. tularensis* subsp. *holarctica* (Jellison type B). *F. tularensis* type A is endemic in North America and type B is located in Europe, Asia, and North America [2-4]. Three biotypes of the less virulent type B have been described: biovar I (erythromycin sensitive), biovar II (erythromycin resistant), and biovar *japonica* which can ferment glycerol [4].

In Germany, human infections are usually caused by skinning, preparing or eating infected hares or drinking contaminated water. *F. tularensis* was sporadically diagnosed in humans in the first half of the 20th century in Germany but almost disappeared in the following decades [5,6]. Between 1983 and 1992 only four sporadic cases of tularemia were notified in hares or rabbits from Lower Saxony, Rhineland-Palatinate, North Rhine-Westphalia and Baden-Württemberg, respectively [6]. After years without reported cases in animals the re-emergence of tularemia started in 2004 with an outbreak of tularemia in a semi-free living group of marmosets (*Callithrix jacchus*) in Lower Saxony [7], and in December 2005 an outbreak with 15 human cases due to contact with infected hares was reported from Hesse [8]. The detection of *F. tularensis* subsp. *holarctica* in organ samples of these hares using PCR assays was the beginning of our investigations of tularemia in European brown hares (*Lepus europaeus*) in Germany.

A variety of PCR methods has been established for the detection of *F. tularensis* DNA in both clinical and environmental specimens [9-11]. Farlow et al. developed a typing assay based on the variable-number of tandem repeats (VNTRs) [12] and Johansson et al. also described a twenty-five VNTR marker typing system that was used to determine the worldwide genetic relationship among *F. tularensis* isolates [1]. Byström et al. selected six of these 25 markers that were highly discriminatory in a study of tularemia in Denmark [13]. Vogler et al. [14] investigated the phylogeography of *F. tularensis* in an extensive study based on whole-genome single nucleotide polymorphism (SNP) analysis. From almost 30,000 SNPs identified among 13 whole genomes 23 clade- and subclade-specific canonical SNPs were identified and used to genotype 496 isolates. This study was expanded upon in another study that used a combination of insertion/deletions (INDELs) and single nucleotide polymorphism analysis [15].

The aim of this study was to elucidate the molecular epidemiology of *F. tularensis* in European brown hares in Germany between 2005 and 2010. Several previously published typing markers were selected and combined in a pragmatic approach to test whether they are suitable to elucidate the spread of tularemia in Germany. This included cultivation, susceptibility testing to erythromycin, a PCR assay for subspecies differentiation detecting a 30 bp deletion in the Ft-M19 locus, VNTR typing, INDEL, SNP, and MALDI-TOF analysis. This is important because it improves our understanding of the spread of tularemia and may help to recognize outbreaks that are not of natural origin.

## Results

### Cultivation and identification of isolates

Cultivation of bacteria from organ specimens was successful in 31 of 52 hares which had a positive PCR result targeting the locus Ft-M19 that was also used to differentiate *F. tularensis* subsp. *holarctica* from other *F. tularensis* subsp. [11]. *F. tularensis* subsp. *holarctica* was identified in all 52 cases.

### Biovars

**Figure 1:**
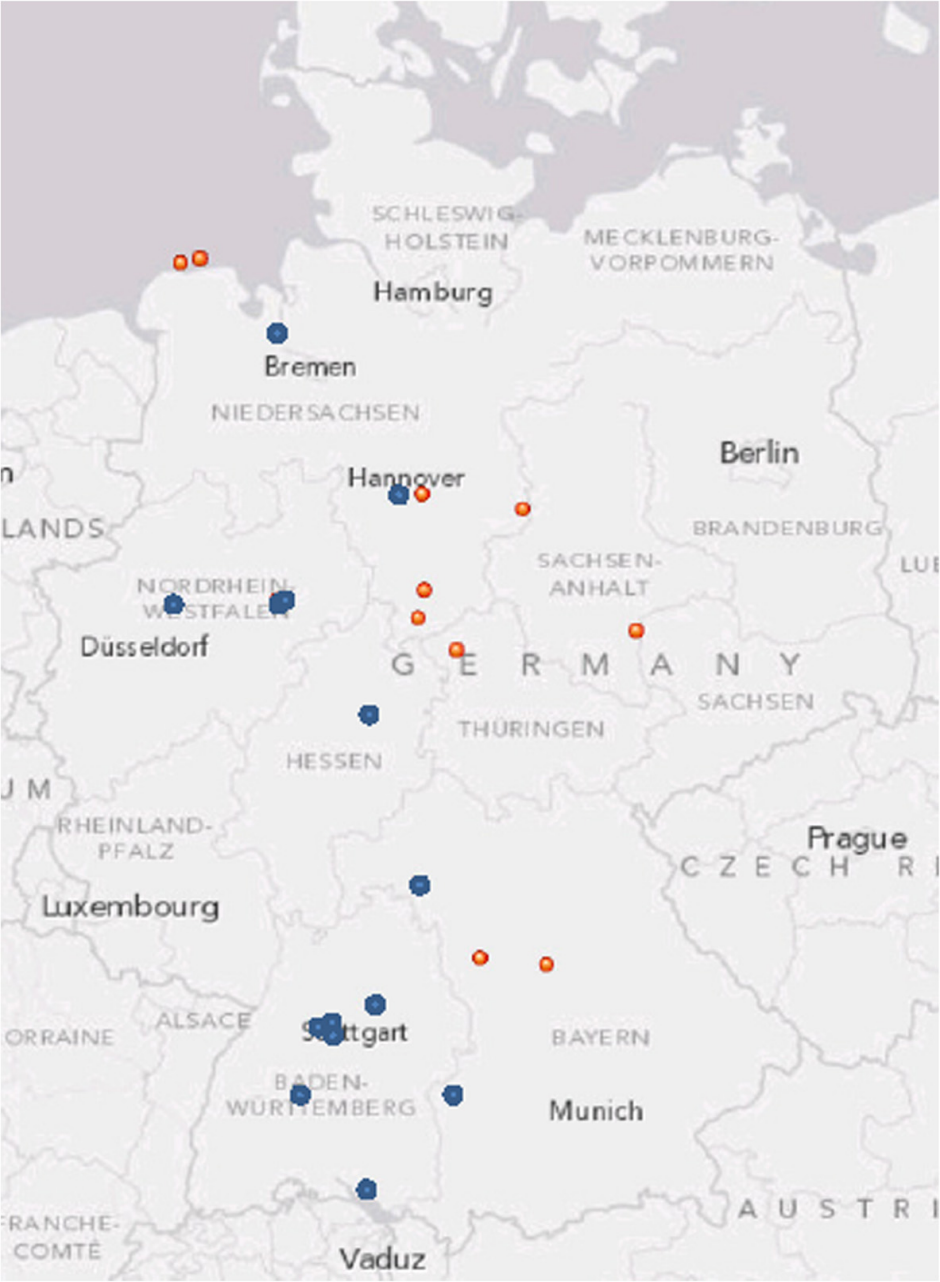
**Germany: areas where *Francisella tularensis *subsp. *holarctica *isolates were found in hares.** Erythromycin sensitive strains occur in regions marked with blue dots, erythromycin resistant strains occur in regions marked with red dots.

Seventeen isolates were susceptible to erythromycin corresponding to biovar I, whereas fourteen were resistant (biovar II). The geographic distribution is given in Table 1, Figure 1 and the susceptibility of the isolates in Additional file 1: Table S2.

**Table 1:** **Original and** g**eographic data of *Francisella tularensis *subsp. *holarctica *isolates (BW – Baden-Württemberg, BY - Bavaria, NRW – North Rhine-Westphalia, LS – Lower Saxony, SN – Saxony, TH - Thuringia)**

**Year**	**Strain number**	**Site**	**Federal state**	**Latitude** **[°NORTH]**	**Longitude [°EAST]**	**Altitude [m]**
2006	06T0001	Moorgrund	TH	50,838005	10,291767	279
2007	08T0001	Dingelstädt	TH	51,315205	10319329	335
2007	08T0008	Allersberg	BY	49,251389	11,234261	388
2007	08T0010	Sehnde	LS	52,31262	9,967105	71
2007	08T0013	Ehingen	BY	49,300734	10,571476	415
2008	08T0014	Weissach-Flacht	BW	48,833991	8,91309	406
2008	08T0015	Leonberg-Höfingen	BW	48,816676	9,016877	379
2007	08T0070	Einbeck-Kohnsen	LS	51,707717	10,000538	121
2008	08T0071	Brake	LS	53,326329	8,478167	2
2008	08T0072	Göttingen-Roringen	LS	51,532638	9,92816	153
2008	08T0073	Twülpstedt-Rümmer	LS	52,224403	11,01102	127
2008	08T0075	Würzburg	BY	49,794256	9,927489	173
2009	09T0105	Geseke	NRW	51,639416	8,509738	105
2009	09T0108	Geseke	NRW	51,639416	8,509738	105
2009	09T0109	Geseke	NRW	47,724358	9,406025	518
2009	09T0114	Markdorf	BW	51,639416	8,509738	105
2009	09T0116	Geseke	NRW	51,444502	12,169177	115
2009	09T0146	Wiedemar	SN	48,864962	9,024819	337
2008	09T0163	Hemmingen	BW	53,770141	7,693722	1
2008	09T0164	Spiekeroog	LS	53,770141	7,693722	1
2008	09T0165	Spiekeroog	LS	53,770141	7,693722	1
2008	09T0166	Spiekeroog	LS	53,770141	7,693722	1
2008	09T0167	Spiekeroog	LS	53,770141	7,693722	1
2008	09T0169	Spiekeroog	LS	53,745892	7,480842	4
2008	09T0171	Langeoog	LS	53,745892	7,480842	4
2009	09T0179	Langeoog	LS	53,745892	7,480842	4
2010	10T0014	Hemmingen	BW	52,314054	9,722783	56
2010	10T0115	Waltrop	NRW	51,624087	7,39465	69
2010	10T0125	Geseke	NRW	51,639223	8,469223	103
2010	10T0128	Empfingen	BW	48,391957	8,708282	491
2010	10T0131	Oppenweiler	BW	48,985678	9,460219	270

### VNTR typing

In a pilot study, six loci (Ft-M3, Ft-M6, Ft-M20, Ft-M21, Ft-M22, and Ft-M24) were amplified and sequenced, but only the loci Ft-M3, Ft-M6, and Ft-M24 were discriminatory. The strains tested in the pilot study are indicated in Additional file 1: Table S2(*). The following identical results were obtained for all these strains: Ft-M20: 255 bp; Ft-M21: 403 bp; Ft-M22: 241 bp. The loci Ft-M3 and Ft-M6 (repeat: TTG GTG AAC TTT CTT GCT CTT) were further used to analyse DNA samples extracted from cultivated bacteria. Sequencing of Ft-M3 identified two different repetitive elements, Ft-M3a (ATC CTT ATT), and Ft-M3b (GTC TTT GTT), respectively. The number of these repeats was determined separately. The size obtained for Ft-M24 (repeat: ATA AAT TAT TTA TTT TGA TTA) correlated with the size observed previously for the B.IV (B.18) clade. All VNTR results are given in Additional file 1: Table S2.

### INDEL analysis

Conventional PCR assays with subsequent gel electrophoretic size determination of the amplicon allowed clear discrimination between amplicons with or without the respective deletions which was confirmed by sequencing in some cases (data not shown). The 31 isolates showed four different INDEL patterns (Additional file 1: Table S2). Based on INDELs and SNPs (see below) 14 isolates were assigned to clade B.I (B.20), one to B.II (B.17), and 16 to B.IV (B.18), according to the nomenclature in Karlsson et al. 2013 [16], where the B.I clade was re-defined to include both B1 and B3 in Svensson et al. [15].

### SNP typing

The results of SNP typing are given in Additional file 1: Table S2. All strains used in a pilot study showed ancestral states of the following SNPs: B.16:G; B.21:G; B.22:G; B.24:T. These strains are indicated (*) in Additional file 1: Table S2. Therefore, only the SNPs B.17, B.18, B.19, and B.20 were further investigated for all isolates.

### MALDI-TOF MS analysis

**Figure 2:**
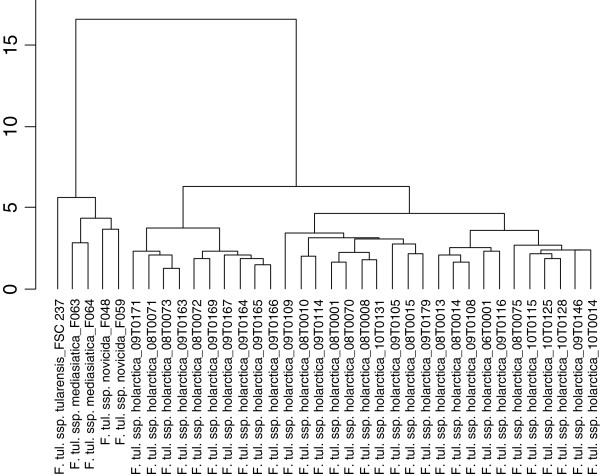
**Dendrogram constructed from MALDI-TOF mass spectrometry spectra of 31 *Francisella tularensis *ssp. *holarctica *strains and representatives of ssp. *tularensis*, *mediasiatica, *and *novicida* .**

All isolates (n=31) yielded high quality spectra. MALDI-TOF was found to be useful for rapid identification of isolates to subspecies level within one hour. However, the obtained clusters (Figure 2) did not conform to the genetic clusters (Additional file 1: Table S2).

### Geographical clustering

Cases of tularemia in hares were identified in eight of sixteen federal states of Germany reaching from islands in the North Sea to regions at Lake Constance in the southern part of Germany. All cases were found below 500 m above sea level. Isolates belonging to biovar I could be found in the western part of Germany whereas biovar II occurred in the eastern region (Table 1 and Additional file 1: Table S2, Figure 1). Molecular typing resulted in further discrimination of clusters within the biovars. Isolates resistant to erythromycin and genetically assigned to clade B.I were found only in Lower Saxony, Thuringia, Bavaria and Saxony. Strains that were sensitive to erythromycin could be assigned to clade B.II (Ftind38) and B.IV (B.18) as given in Additional file 1: Table S2.

### Stability testing

The investigated markers for two *Francisella* isolates (06T0001 from hare and 10T0191 from fox) were stable even after 20 passages in cell culture and had identical results for the markers Ft-M3 (297 bp), Ft-M6 (311 bp), Ftind33 (deletion), Ftind38 (insertion), and Ftind49 (insertion).

## Discussion

In Thuringia the first case of tularemia in a hare was reported in 2006 [17]. In Lower Saxony 2,162 European brown hares and European rabbits (*Oryctolagus cuniculus*) were screened for tularemia between 2006 and 2009 using cultivation and PCR assays. *Francisella* specific PCR assays were positive in 23 hares and 1 rabbit which were further confirmed by cultivation of *F. tularensis* subsp. *holarctica* in 12 hares [18]. In the present study, cases of tularemia in hares in Germany from 2005 to 2010 were investigated. During this period a total of 52 hares were found positive in PCR assays for *F. tularensis* subsp. *holarctica* DNA and from 31 of these cases *Francisella* strains could be isolated. MALDI-TOF analysis was also used to rapidly identify *Francisella* to the subspecies level as was previously shown by Seibold et al. [19].

Several positive specimens were found on the North Sea islands Langeoog and Spiekeroog (LS), around Soest (NR), Darmstadt (H), and Böblingen (BW). These natural foci and also sporadic cases in other regions of Germany were found below 500 m above sea level. In the Czech Republic typical natural foci of tularemia occurred in alluvial forests and field biotopes below 200 m sea level with mean annual air temperature between 8.1-10.0°C and mean annual precipitation of 450–700 mm [20]. In Germany, an outbreak of tularemia in a colony of semi-free living marmosets was located in a region with geographic and ecological conditions similar to the hare habitats in the Czech Republic: field biotopes 175 m above sea level (<200 m) with 9.2°C mean annual air temperature and 642 mm mean annual precipitation [8]. In Germany, tularemia of hares occurs in regions with rather humid soil like in alluvial forests and alongside rivers, but this obviously corresponds with the natural habitat of hares.

Specimens were screened using a PCR assay targeting Ft-M19 described by Johansson et al. [11] which allows the simultaneous identification of the species *F. tularensis* and the differentiation of the subspecies *holarctica* from other (sub-) species. All samples could be attributed to *F. tularensis* subsp. *holarctica*.

We found a clear segregation of clade B.I and clade B.IV in Germany, B.I strains dominate in eastern Germany and B.IV within western Germany (Figure 1). Clade B.I is known to dominate in Europe between Scandinavia and the Black Sea [15,16,21-23]. The other dominating European clade is B.IV (B.18) which can be found over a large area of western and central Europe, and, based upon this study, western Germany [21,23-26]. We found only one strain of the B.II clade isolated in Bavaria. Strains of the B.II clade are most frequently isolated in the USA, but are found sporadically in Europe as well [16,21].

The phylogeographical pattern of clade B.I and B.IV, coincide with the geographical distribution of biovar II and biovar I strains, respectively. Previously, biovar I strains (erythromycin sensitive) have been reported from Western Europe (France, Germany, Spain and Switzerland), North-America, Eastern Siberia and the Far East while biovar II is present in the European part of Russia as well as Northern, Central and Eastern Europe (Austria, Germany, Sweden and Turkey) [27-31]. A mixture of both biotypes has been reported in Sweden, Norway, Bulgaria, Russia and Kazakhstan [27,28,32]. Isolation of both biovars from rodents in a single settlement in Moscow as well as from water samples collected in the Novgorod region [27] indicate coexistence of the biovars in the same epidemiological foci. Taken together, a geographical separation of *F. tularensis* strains seems to exist in Germany. The phenotypically defined biovar I (erythromycin sensitive) and phylogenetically defined clade B.IV strains are confined in western Germany, whereas biovar II (erythromycin resistance) and clade B.I strains cluster in eastern Germany. This is interesting and may reflect a competition between the two subpopulations or unknown underlying ecological or epidemiological differences.

A deletion in the genome of *F. tularensis* subsp. *holarctica* in RD_23_ is typical for strains of *F. tularensis* subsp. *holarctica* in France, the Iberian Peninsula and also Switzerland, where biovar I predominates [24,25,27]. However, in one erythromycin susceptible isolate from Bavaria (08T0013), classified in this study as belonging to the B.II clade, RD_23_ was not deleted, thus showing that deletion of RD_23_ is not correlated with sensitivity to erythromycin. The molecular mechanisms of resistance to erythromycin have not been functionally established, but mutations identified in domain V of the 23S rRNA of biovar II strains, could provide a likely explanation [33].

Although 25 VNTR markers have been described for the typing of *Francisella*, it is pragmatic to investigate only loci of interest depending on the prevalent subspecies of *F. tularensis,* the efficiency of PCR assays for single loci, and existing data [1,13,34]. Sequence analysis of the locus Ft-M3 resulted in two different repeats denominated here as Ft-M3a corresponding with SSTR9E and Ft-M3b corresponding with SSTR9A as described previously by Johansson et al. [35]. Johansson et al. and Byström et al. also found that locus Ft-M3 is the most variable marker [1,13]. In the *Francisella* genome variations of DNA sequences in spite of identical repeat length have been described for short-sequence tandem repeats [35,36]. Locus Ft-M6 showed less variability with only three PCR fragment sizes being observed among the strains. We obtained the same amplicon sizes that were described in previous studies for locus Ft-M3 (Additional file 1: Table S2) [14,37] and for locus Ft-M6 (Additional file 1: Table S2) [14,37]. Svensson et al. developed a sophisticated real-time PCR array for hierarchical identification of *Francisella* isolates [15]. Only three (Ftind33, Ftind38, Ftind49) out of five INDEL loci were discriminatory among our set of *F. tularensis* subsp. *holarctica* isolates. Ftind48 is a marker for B.I to B.IV clades (non-japonica/non-california) and is not expected to vary for these isolates, and Ftind50 is targeting a specific deletion that so far only has been found in LVS. It was possible to simplify these assays to conventional PCR assays that allowed a simple read out based on gel electrophoresis. We identified clusters of strains that had the same INDELs and SNPs as strains described by Svensson et al. [15]. In our study the analysis of VNTR and INDELs of two *F. tularensis* subsp. *holarctica* strains (06T0001, 10T0191) that were passaged twenty times in Ma-104 cells showed that these genomic elements were stable. Johansson et al. demonstrated for two VNTR loci (SSTR9 and SSTR16) that they were actually stable over 55 passages [35]. The VNTR pattern for strains belonging to clade B.I was more variable compared with the pattern obtained for clade B. IV (Additional file 1: Table S2), as was observed previously [21,23-25]. This might indicate that clade B.IV is more recently introduced in Germany than clade B.I.

We have applied several typing tools in a polyphasic approach in order to determine their value for identifying groups of *Francisella* strains in Germany. We found strains belonging to biovars I and II of *F. tularensis* subsp*. holarctica*. Although SNP loci are the most informative markers for typing of *Francisella* this method may have to be adapted to local strains [37,38].

## Conclusions

*F. tularensis* seems to be a re-emerging pathogen in Germany that infects hares in many regions and causes a potential risk for exposed humans such as hunters and others who process hares. The pathogen can easily be identified using PCR assays directly on DNA extracted from organ specimens or cultivated strains. Isolates can also be identified rapidly using MALDI-TOF MS in routine laboratories where specific PCR assays for *F. tularensis* are not established. To identify differences and genetic relatedness of *Francisella* strains, analysis of VNTR loci (Ft-M3, Ft-M6 and Ft-M24), INDELs (Ftind33, Ftind38, Ftind49, RD23) and SNPs (B.17, B.18, B.19, and B.20) was shown to be useful in this set of strains. When time and costs are limiting parameters isolates can be analysed using simplified PCR assays with a focus on genetic loci that are most likely discriminatory among strains found in a specific area. For the future whole genome sequencing using next generation sequencing is desirable and should provide more genetic information of *Francisella* strains. Based on these data a more detailed view on the epidemiology of tularemia will become possible [39].

## Methods

### Samples

Organ specimens (e.g. spleen, liver, lung, and/or kidney) of European brown hares that were suspicious of tularemia were collected by local veterinary authorities in Germany since 2005 and sent for confirmatory testing to the National Reference Laboratory for Tularemia of the Friedrich-Loeffler-Institut in Jena. *Francisella* strains were cultivated on cysteine heart agar (Becton Dickinson GmbH, Heidelberg, Germany) supplemented with 10% chocolatized sheep blood and antibiotics in order to suppress the growth of contaminants. One litre of culture medium contained 100 mg ampicillin (Sigma-Aldrich Chemie, Taufkirchen, Germany) and 600 000 U polymyxin B (Sigma-Aldrich Chemie). Plates were incubated at 37°C with 5% CO_2_ for up to 10 days. Typical colonies are grey-green, mostly confluent, glossy, and opaque. Gram staining was performed routinely and showed Gram negative coccoid bacteria.

The reference strains *F. tularensis* subsp. *tularensis* (FSC 237), *mediasiatica* (FSC 147), and *F. novicida* (ATCC 15482) were obtained from the Bundeswehr Institute of Microbiology, Munich, Germany, and *F. philomiragia* (DSMZ 7535) was obtained from the German Collection of Microorganisms and Cell Cultures, Braunschweig, Germany, respectively.

### Erythromycin susceptibility

All *F. tularensis* subsp. *holarctica* isolates were tested for their erythromycin susceptibility using Erythromycin discs [30 μg] and M.I.C.Evaluator™ (Oxoid, Wesel, Germany) in order to discriminate the susceptible biovar I from the resistant biovar II as described previously [40].

### DNA extraction

50 mg of organ material or 200 μl of cell culture supernatant were lysed and the DNA was extracted using the High Pure PCR Template Preparation Kit (Roche Diagnostics, Mannheim, Germany) according to the manufacturer`s instructions. If cultivation was successful some colonies were resuspended in 200 μl phosphate-buffered saline, boiled at 90°C for 10 minutes and DNA was prepared as described above. Finally, DNA was eluted in 200 μl elution buffer. 5 μl were applied in each PCR assay.

### Diagnostic PCR assay

*F. tularensis* subsp. *holarctica* was identified using a PCR assay with primer pair C1/C4 targeting the locus Ft-M19 that distinguishes the two major subspecies *F. tularensis* subsp. *holarctica* and *F. tularensis* subsp. *tularensis* which was carried out as described by Johansson et al. [11].

### VNTR typing

In pilot experiments 6 VNTR loci (Ft-M3, Ft-M6, Ft-M20, Ft-M21, Ft-M22, and Ft-M24) were investigated as described by Byström et al. [13]. The loci found discriminatory were then subsequently analysed in all 31 isolates. The amplification of the VNTR loci was carried out under the same cycling conditions as the diagnostic PCR assay except for the annealing temperature of 56°C. The fragments were cut out of the agarose gel and DNA was purified using the innuPrep Gel Extraction Kit (Analytik Jena AG, Jena, Germany) according to the manufacturer’s instructions. Subsequently, DNA amplificates were sequenced as described below.

### INDEL analysis

Five INDELs (Ftind33, Ftind38, Ftind48, Ftind49, and Ftind50) that are discriminatory among *F. tularensis* subsp. *holarctica* were selected from the loci described by Svensson et al. [15]. The real-time PCR assays with melting curve analyses were simplified by using conventional PCR assays. The primers “CP” and “OUT” for the respective loci were used as described by Svensson et al. The reaction mixture consisted of 5 μl 10 x PCR buffer with 1.5 mM MgCl_2_ (Genaxxon, Stafflangen, Germany), 2 μl of dNTP mix (each 2 mM, Carl Roth GmbH, Karlsruhe, Germany), 1 μl of each primer, 0.2 μl of *Taq* DNA polymerase (5 U/μl, Genaxxon), 5 μl of DNA extract and deionised water to a final volume of 50 μl. After denaturation at 95°C for 5 min, 35 cycles of amplification were performed with denaturation at 95°C for 30 s, primer annealing at 60°C for 60 s, and primer extension at 72°C for 30 s. After a final extension step at 72°C for 30 s amplicons were separated using 2.5% agarose gel electrophoresis and visualized using ethidium bromide staining under UV light.

### SNP typing

Four of ten SNPs (B.17, B.18, B.19, and B.20) that have been found to be useful for the typing of *F. tularensis* subsp. *holarctica* strains were selected from the loci described by Svensson et al. [15]. The primers “C” and “D” for the respective loci described by Svensson et al. were used, but the primers “D” were shortened by removing the SNP specific last nucleotide and the non-binding GC-rich tails that were originally added to the allele-specific primer (i.e. gcgggcagggcggc). SNPs were detected by sequence analysis of the PCR products. The nomenclature used for the designation of clades is according to Karlsson et al. 2013 [16].

### DNA sequencing

Purified DNA fragments were subjected to cycle sequencing with BigDye™ Terminator Cycle Sequencing Ready Reaction Kit (Applied Biosystems, Darmstadt, Germany). Amplification primers were also used as sequencing primers. Nucleotide sequences were determined on an ABI Prism 310 Genetic Analyzer (Applied Biosystems).

### Analysis of sequence data

VNTR sequence data were aligned using BioEdit (Biological sequence alignment editor, Ibis Therapeutics, Carlsbad, CA, USA).

### Stability testing

The stability of the markers Ft-M3, Ft-M6, Ftind33, Ftind38, and Ftind49 was assessed for two *F. tularensis* subsp. *holarctica* strains that were isolated from a hare (06T0001) and a red fox (*Vulpes vulpes*) (10T0191), respectively. The isolates were passaged twenty times on MA-104 cells in 12.5 ml cell culture flasks (Becton Dickinson GmbH, Heidelberg, Germany). Confluent monolayers of MA-104 cells were washed with phosphate- buffered saline, pH 7.4. The bacterial suspensions or cell culture samples were inoculated on the cells at 37°C for 1 h. The inoculum was replaced with Dulbecco’s Modified Eagle’s Medium (DMEM) and incubated at 37°C in a humidified air atmosphere with 5% CO_2_. After incubation for 3 to 5 days when the cells detached from the surface, the bacteria were harvested by two freeze-thaw cycles. The bacteria/cell suspensions were used for preparation of DNA.

### MALDI-TOF typing

Samples were taken from single colonies, ethanol-precipitated and extracted with 70% formic acid as described by Sauer et al. [41]. The extract was diluted with one volume acetonitrile and 1.5 μL of the mixture was spotted to a steel MALDI target. The dried extract was overlaid with 1.5 μL of a saturated solution of α-cyano-4-hydroxycinnamic acid in 50% acetonitrile/2.5% trifluoroacetic acid as matrix and was again allowed to dry. A custom-made database of reference spectra, or main spectra (MSP), was constructed using the BioTyper software (version 1.1, Bruker Daltonics, Bremen, Germany) following the guidelines of the manufacturer. Each sample was spotted six-fold and four single spectra with 500 laser pulses each were acquired from each spot with an Ultraflex I instrument (Bruker Daltonics) in the linear positive mode in the range of 2,000 to 15,000 Da. Acceleration voltage was 25 kV and the instrument was calibrated in the range of 4,364 to 10,299 Da with reference masses of an extract of an *Escherichia coli* DH5-α strain prepared according to Sauer et al. [41]. MSP were generated within the mass range of 2,500 to 15,000 Da with the following default parameters: compression of the spectrum data by a factor of 10, baseline smoothing by the Savitsky-Golay algorithm (25 Da frame size), baseline correction by 2 runs of the multi-polygon algorithm, and peak search by spectra differentiation. The number of peaks was limited to 100 per MSP and all peaks were normalized to the most intense peak with an intensity of 1.0. The minimum frequency of occurrence within the 24 single spectra was set to 50% for every mass. Peak lists of MSP were exported for further evaluation.

## Competing interests

The authors declare that they have no competing interests.

## Authors’ contributions

WM participated in the design of the study, evaluated VNTR data and drafted the manuscript. HH performed PCR assays and DNA sequencing and critically revised the manuscript. PO performed cultivation on nutrient agar and cell culture, erythromycin susceptibility testing, and critically revised the manuscript. AK performed MALDI-TOF MS experiments, data analysis and drafted the respective sections in the manuscript. BB performed MALDI-TOF MS experiments and data analysis. HB isolated and cultivated strains and critically revised the manuscript. SB performed *post mortem* examination and bacterial culture and revised the manuscript. UE performed *post mortem* examination and bacterial culture and revised the manuscript. SH provided sample specimens and strains and critically revised the manuscript. RK provided sample specimens and strains and critically revised the manuscript. AN performed *post mortem* examination and bacterial culture and revised the manuscript. MP contributed tissues of hares with tularemia from the region of Soest (NRW). MR did PCR assays to identify *Francisella tularensis* in samples and bacterial cultures and revised the manuscript. GS participated in the data analysis and critically revised the manuscript. BAS isolated and cultivated a *Francisella tularensis* strain from European brown hare in Saxony and critically revised the manuscript. RS isolated and cultivated a *Francisella tularensis* strain from European brown hare in Bavaria and critically revised the manuscript. KM participated in the data analysis of typing data and critically revised the manuscript. EK typed strains and critically revised the manuscript. MF participated in the data analysis and critically revised the manuscript. HT participated in the design of the study, coordinated the experiments, analysed the data, and finalized the manuscript. All authors read and approved the final manuscript.

## Supplementary Material

Additional file 1: Table S2 Results of VNTR, SNP, INDEL analysis and erythromycin sensitivity testing of *Francisella tularensis* subsp. *holarctica* isolates. The number of repeats is given for Ft-M3a, Ft-M3b, and Ft-M6. The number of base-pairs is given for Ft-M24. Derived state of SNPs and INDELs is in boldface. Nomenclature is according to Karlsson et al. (2013) [16], where the B.I clade was re-defined to include both B1 and B3 [15] (DEL, deletion; IN, insertion; bp, basepairs; BW – Baden-Württemberg, BY - Bavaria, NRW – North Rhine-Westphalia, LS – Lower Saxony, SN – Saxony, TH – Thuringia; n.d., not done). Click here for file
